# Evaluation of intrafraction couch shifts for proton treatment delivery in head‐and‐neck cancer patients: Toward optimal imaging frequency

**DOI:** 10.1002/acm2.13795

**Published:** 2022-10-14

**Authors:** Nrusingh C. Biswal, Dario B. Rodrigues, Weiguang Yao, Jason K. Molitoris, Matthew E. Witek, Shifeng Chen

**Affiliations:** ^1^ Department of Radiation Oncology University of Maryland School of Medicine Baltimore Maryland USA

**Keywords:** cone‐beam CT, couch shift, H&N, image‐guided radiation therapy, kV imaging, proton therapy

## Abstract

**Purpose:**

Treatment planning for head‐and‐neck (H&N) cancer, in particular oropharynx, nasopharynx, and paranasal sinus cases, at our center requires noncoplanar proton beams due to the complexity of the anatomy and target location. Targeting accuracy for all beams is carefully evaluated by using image guidance before delivering proton beam therapy (PBT). In this study, we analyzed couch shifts to evaluate whether imaging is required before delivering each field with different couch angles.

**Methods:**

After the Institutional Review Board approval, a retrospective analysis was performed on data from 28 H&N patients treated with PBT. Each plan was made with two‐to‐three noncoplanar and two‐to‐three coplanar fields. Cone‐beam computed tomography and orthogonal kilovoltage (kV) images were acquired for setup and before delivering each field, respectively. The Cartesian (longitudinal, vertical, and lateral) and angular (pitch and roll) shifts for each field were recorded from the treatment summary on the first two fractions and every subsequent fifth fraction. A net magnitude of the three‐dimensional (3D) shift in Cartesian coordinates was calculated, and a 3D vector was created from the 6 degrees of freedom coordinates for transforming couch shifts in the system coordinate to the beam's‐eye view.

**Results:**

A total of 3219 Cartesian and 2146 angular shift values were recorded for 28 patients. Of the Cartesian shifts, 2069 were zero (64.3%), and 1150 (35.7%) were nonzero (range, −7 to 11 mm). Of the angular shifts, 1034 (48.2%) were zero, and 1112 (51.8%) were nonzero (range, −3.0° to 3.2°). For 17 patients, the couch shifts increased toward the end of the treatment course. We also found that patients with higher body mass index (BMI) presented increased net couch shifts (*p* < 0.001). With BMI < 27, all overall net shift averages were <2 mm, and overall maximum net shifts were <6 mm.

**Conclusions:**

These results confirm the need for orthogonal kV imaging before delivering each field of H&N PBT at our center, where a couch rotation is involved.

## 1 INTRODUCTION

Image‐guided radiation therapy (IGRT) has been the standard of care for treating head‐and‐neck (H&N) cancers. Among all radiation treatment techniques, intensity‐modulated proton therapy (IMPT) offers great benefit to H&N patients because of the sharp dose falloff and use of multifield optimization (MFO) algorithms that help spare critical healthy organs while treating targets with high conformality.^1^, ^2^, ^3^, ^4^ Several studies have suggested that IMPT has added value for H&N squamous cell carcinoma, because it is more effective than intensity‐modulated (photon) radiation therapy (IMRT) in reducing radiation treatment side effects.^5^ However, the physical properties of protons make IMPT more sensitive than photons to planning uncertainties.^6^, ^7^ Because of the high sensitivity of proton dose distribution to the patient setup, IGRT setup becomes the ultimate choice to treat each fraction of the treatment plan.^4^, ^8^, ^9^, ^10^ In‐room and onboard imaging methods obtain information on target position and movement in intra‐ and interfractions, compare these images with reference imaging taken initially, and give feedback to optimize patient setup and target localization. Radiation oncology incident learning systems have demonstrated that incorrect or omitted patient shifts during treatment are common near misses or incidents in radiation therapy.^11^ Because protons are quite sensitive to small changes in tissue density along the beam's path, weight loss and tumor response (common in H&N treatment) require diligent monitoring of interactional change for optimal patient management.^7^, ^12^, ^13^ To this end, a robust clinical setup is required during the course of proton beam treatment of H&N cancer patients.

H&N patients are planned with multiple coplanar and noncoplanar proton beams because of the complexity of anatomy, irregularly shaped targets, and the proximity of targets to numerous organs at risk (OARs).^14^ This complexity requires careful delivery of each treatment field. Thus, at our center imaging frequency and accuracy requirements for the couch position and gantry angles increase for these patients. Targeting accuracy at all possible couch and gantry angles must be carefully evaluated, at our center, prior to delivering proton beams. As a result, patients are on the table for much longer during IMPT than for standard radiation treatment procedures (e.g., IMRT). This increased table time may add intrafractional patient movement. Hence, there is a need to evaluate the necessity of intrafractional IGRT and find the couch shifts while going from one field to the next. Several studies investigated the setup accuracy comparison based on cone‐beam computed tomography (CBCT) and kilovoltage (kV) images.^10^, ^12^, ^13^, ^15^, ^16^, ^17^ Kraan et al.^7^ found increased target dose deterioration with increasing setup errors by introducing a systematic shift of isocenter by 2 mm. They also found that the treatment intent of 90% of population to have *D*98% > 95% of the prescription dose was no longer satisfied. The largest *D*98% spread in clinical tumor volume (CTV)‐66 Gy was seen in relatively small tumors. In an intracranial stereotactic radiotherapy (SRT) study, Tanaka et al. found that patient position errors were detected after couch rotation while using noncoplanar beams. These position errors exceeded the planning target volume (PTV) margin of 1.0–2.0 mm that is commonly used in SRT.^15^ In another study, Ye et al. investigated the daily setup error in prostate IGRT with fiducial‐based kV and CBCT imaging and found that the majority (79%) of patients presented a net residual three‐dimensional (3D) shift <3 mm. However, to the best of our knowledge, there are no comparisons of shifts between orthogonal kV and CBCT images for proton beam treatment of H&N patients with noncoplanar beams. This warrants a thorough analysis of CBCT and kV images acquired during patient setup to establish an image‐guided setup procedure of these treatments. Note that our robust plans adopt a value for setup uncertainty on CTV, which is somewhat equivalent to PTV margin in non‐robust planning.

In this retrospective study, we analyzed CBCT and kV images acquired before delivering proton beams in a group of 28 H&N patients of bilateral oropharynx, nasopharynx, and paranasal sinus cases. We evaluated the shifts to determine their magnitude to aid in image guidance recommendations. This analysis is critical in understanding the necessity for imaging before delivering each field for patients undergoing H&N image‐guided proton beam radiotherapy.

## 2 MATERIALS AND METHODS

After the Institutional Review Board approval, treatment data from 28 patients (24 men, 4 women; median age, 63 years; range, 44–82 years) were reviewed. The patients were treated in 2019 at our institution with total doses of 6000–7000 cGy in 30–50 fractions with fraction doses of 120–212.1 cGy.

### 2.1 Treatment planning

Simulation scans were performed on a Siemens Somatom Definition Edge CT scanner (Siemens Healthineers; Erlangen, Germany) in the head‐first supine position and with Qfix 5‐point mask and base‐of‐skull frame (Qfix; Avondale, PA). Axial images were obtained every 1.5 mm from one vertebral body below the carina inferiorly to the top of the skull superiorly. Tentative isocenter (or coordinate reference) positions were marked with radiopaque markers.

Structure delineation and treatment planning were performed in a RayStation 8A (RaySearch Laboratories; Stockholm, Sweden) treatment planning system. To improve dose delivery robustness, our institute's policy requires that any part of the target is covered by at least two beams with no beam contributing >70% of the prescription dose. With this policy, for bilateral diseases our IMPT plans typically had two‐to‐three coplanar and two‐to‐three noncoplanar spot‐scanning (4‐mm spot size) proton beams. Each plan was optimized using the MFO technique to achieve target coverage goals of 100% of the gross tumor volume and 95% of the CTV receiving at least 100% of the prescription dose, while maintaining OAR doses below the constraints of our institutional practice guidelines. We attempted to spare OARs as long as coverage was not sacrificed. The two‐to‐three coplanar beams were oriented at gantry angles of one beam at 0°, and the other one‐to‐two beam(s) within 150°–210° (posterior–anterior [PA] and posterior–oblique beams). The two‐to‐three noncoplanar beams were oriented at gantry (G) and couch (C) angle combinations (G_C) of 60°–75°_340°–345°, 285°–300°_10°–20°, and 295°–335°_60°–90°. Couch angles of around 15° were used for anterior–oblique fields to avoid the beam passing through the shoulders. The anterior–posterior beam usually covered the anterior part of the target from the inferior portion of the mandible. The PA beam covered all parts of the targets except those in the brain and lungs. In certain complex cases, the PA beam was sometimes treated through the lungs and brain. The beam 295°–335°_60°–90° was used to reduce brainstem dose. We used both simultaneous integrated boost (SIB) and sequential treatments, depending on individual case requirements.

The RayStation treatment planning system Monte Carlo–based dose optimization and dose calculation engine were used to generate treatment plans. Plans were robustly optimized, using CTVs as target volumes and accounting for a 3‐mm setup uncertainty in lateral, longitudinal, and vertical directions and 3.5% for range uncertainty, with 12 worst case scenarios. In the robust analysis, we required at least *D*95% > 95% of the prescription dose for the worst scenario in setup (Cartesian shift but not rotation) and range uncertainties. The independent beam optimization was not utilized for these plans. The layer spacing was dependent on whether there was a range shifter and the type of range shifter used in the plan. A 3‐cm range shifter was utilized in which the relative scale of the spot and layer spacing was manipulated to ensure that the spacing for both aspects fell within the range of 0.5–0.9 cm for both spot and layers. OARs utilized for robust optimization were the serial organs, such as spinal cord, brainstem, and optic apparatus. The beam angles were chosen to spread out the end‐of‐range effect for OARs to spread the impact of the distal relative biological effectiveness.

We consider planning more robustly for unilateral targets that are far away from OARs when no prior radiation was involved. However, for bilateral cases, we need to compromise among adequate coverage, robustness, and OAR sparing. If our bilateral cases were planned more robustly, we would potentially sacrifice OAR robustness for serial organs. We prioritize OAR robustness for serial organs for patients who have received prior radiations.

### 2.2 Treatment delivery

**FIGURE 1:**
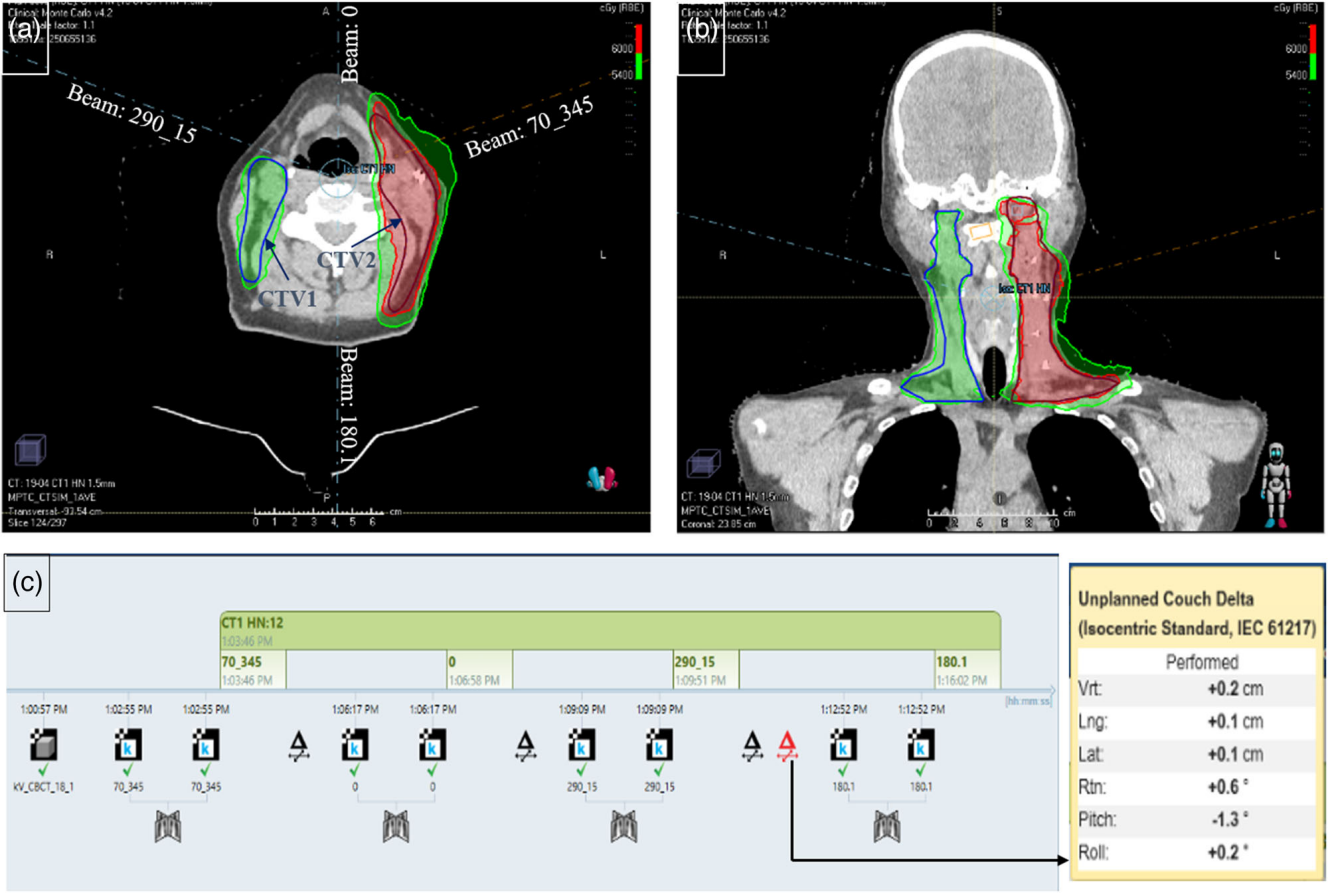
(a) Axial and (b) coronal planes of isodose lines, clinical tumor volumes (CTVs), and beam arrangements in a 64‐year‐old man with malignant neoplasm of tonsillar fossa, diagnosed with oropharyngeal squamous cell carcinoma. (c) Image‐guided radiation therapy (IGRT) beam delivery with couch shift for one of the beams in the inset

Treatments were delivered on a ProBeam (Varian Medical Systems, Inc.; Palo Alto, CA) machine with a 6‐degree‐of‐freedom (6‐DOF) robotic couch. Per our institutional policy, patients were initially set up on the couch with a kV–kV for bony structure matching, then CBCT for bony structure matching and soft tissue checking to ensure the appropriate alignment of the treated volumes with the planning CT, followed by another kV–kV for treatment fusion verification at beam's‐eye view. CBCT may not be performed each fraction, varying case by case. When the couch angle changed during the progression of treatment fields, a kV image was acquired to enable couch adjustment. A representative patient's plan with beam setup, isodose lines, and plan delivery are presented in Figure 1. The patient was a 64‐year‐old man with a malignant neoplasm of the tonsillar fossa, diagnosed with oropharyngeal squamous cell carcinoma. A dose of 5400 cGy was prescribed to CTV1 (right tonsil fossa/base of tongue/soft palate) and 6000 cGy in an SIB technique to the CTV2 (left tonsil, palate, and base of tongue) in 30 fractions using 4 pencil‐beam scanning proton beams (2 coplanar and 2 noncoplanar). The CTVs, isodose lines, and beam arrangements in axial and coronal planes are presented in parts (a) and (b) of Figure 1, respectively. Figure 1c presents the beam delivery sequence, with the couch shift in the insert.

### 2.3 Couch shift data analysis

Cartesian (longitudinal, vertical, and lateral) and angular (yaw, pitch, and roll) shifts for each field were extracted from the treatment summary (ARIA 15.1, Varian Medical Systems, Inc.) for the first two fractions and every fifth fraction thereafter. Based on individual coordinate shifts, a net 3D shift magnitude vector *R* in Cartesian coordinates was calculated as

(1) $$R=\sqrt{\Delta Lng^{2}+\Delta Vrt^{2}+\Delta Lat^{2}},$$

where Δ*Lng*, Δ*Vrt*, and Δ*Lat* represent changes in longitudinal, vertical, and lateral coordinates, respectively, referenced to CBCT. Similarly, angular shifts (Δ*Roll* and Δ*Pitch*) were calculated with reference to CBCT. Collectively, the three Cartesian shifts will be referred to as Δ*r*, and the two angular shifts will be referred to as Δ*θ*.

A 3D vector was created from the 6‐DOF coordinates for transforming the couch shifts in the system coordinate (i.e., originating at beam isocenter and gantry angle = 0°, couch angles = 0°, 0°, 0°) to the beam's‐eye view.

The vector dimensions were defined as

(2) $$\begin{array}{c} \begin{array}{ccc} Vx & = & x\left[\cos\left(Roll\right)\cdot \cos\left(Pitch\right)\right]\\ & & +\;y\left[\cos\left(Roll\right)\cdot \sin\left(Pitch\right)\cdot \sin\left(Yaw\right)-\sin\left(Roll\right)\cdot \cos\left(Yaw\right)\right]\\ & & +\;z\left[\cos\left(Roll\right)\cdot \sin\left(Pitch\right)\cdot \cos\left(Yaw\right)+\sin\left(Roll\right)\cdot \sin\left(Yaw\right)\right] \end{array} \end{array}$$

(3) $$\begin{array}{c} \begin{array}{ccc} Vy & = & x\left[\sin\left(Roll\right)\cdot \cos\left(Pitch\right)\right]\\ & & +\;y\left[\sin\left(Roll\right)\cdot \sin\left(Pitch\right)\cdot \sin\left(Yaw\right)+\cos\left(Roll\right)\cdot \cos\left(Yaw\right)\right]\\ & & +\;z\left[\sin\left(Roll\right)\cdot \sin\left(Pitch\right)\cdot \cos\left(Yaw\right)-\cos\left(Roll\right)\cdot \sin\left(Yaw\right)\right] \end{array} \end{array}$$

(4) $$\begin{array}{ccc} Vz & = & -x\left[\sin\left(Pitch\right)\right]+y\left[\cos\left(Pitch\right)\cdot \sin\left(Yaw\right)\right]\\ & & +\;z\left[\cos\left(Pitch\right)\cdot \cos\left(Yaw\right)\right], \end{array}$$

where *x*, *y*, and *z* represent the Cartesian shifts Δ*Lng*, Δ*Vrt*, and Δ*Lat*, respectively, corresponding to the reference CBCT; *Yaw* is the couch rotation from 0° to the planned couch angle; *Roll* is the gantry rotation from 0° to the beam angle; and *Pitch* is the couch pitch angle. *Vx*, *Vy*, and *Vz* are the coordinates of the couch shift in the beam's‐eye view coordinate system, where the axis for *Vz* matches the beam central axis; and *Vx* and *Vy* are the coordinates on the plane passing the machine isocenter and perpendicular to the beam central axis, respectively. Hence, (*Vx*, *Vy*, *Vz*) is the shift in beam's‐eye view from the couch shift in Cartesian coordinates. All net shifts were averaged over all beams and over all patients at the respective fraction numbers.

A net 3D transformation vector in beam's‐eye view was calculated as

(5) $$V_{3D}=\sqrt{Vx^{2}+Vy^{2}+Vz^{2}}.$$

## 3 RESULTS

**FIGURE 2:**
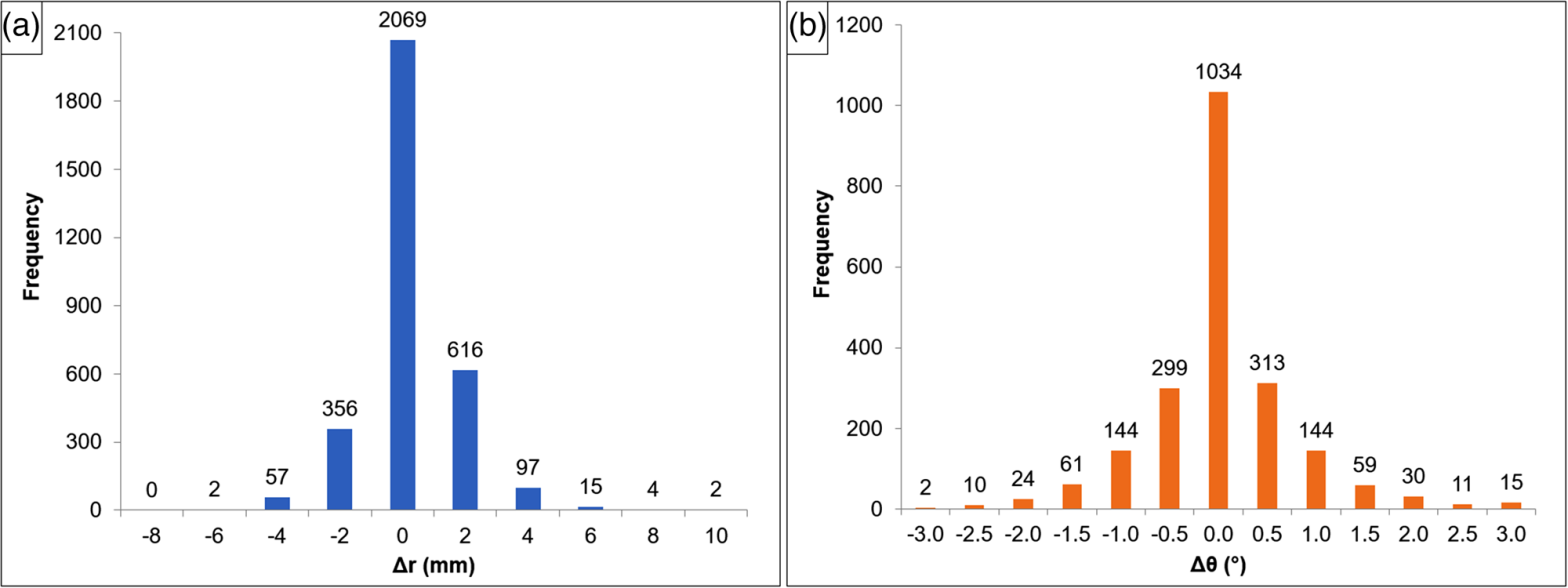
Histogram distribution for couch shifts in 28 patients: (a) Cartesian shifts, where Δ*r* includes the Δ*Lng*, Δ*Vrt*, and Δ*Lat*; and (b) angular shifts in degrees, where Δ*θ* includes Δ*Yaw* and Δ*Pitch*

A total of 3219 Cartesian and 2146 angular shift values were recorded for the 28 patients. The couch rotation (yaw angle) was changed as intended per plan, so we excluded this parameter from the angular shifts and reported the other five shifts: vertical, longitudinal, lateral, pitch, and roll. Out of 3219 Cartesian shifts, 2069 were zero (64.3%), and the remaining 1150 (35.7%) were non‐zero, ranging from −7 to 11 mm. Similarly, of the 2146 angular shifts, 1034 were zero (48.2%), and the remaining 1112 (51.8%) were nonzero, ranging from −3.0° to 3.2°. As shown in Figure 2, the distributions of Cartesian and angular shifts have Gaussian shapes centered at 0 mm and 0°, respectively.

**FIGURE 3:**
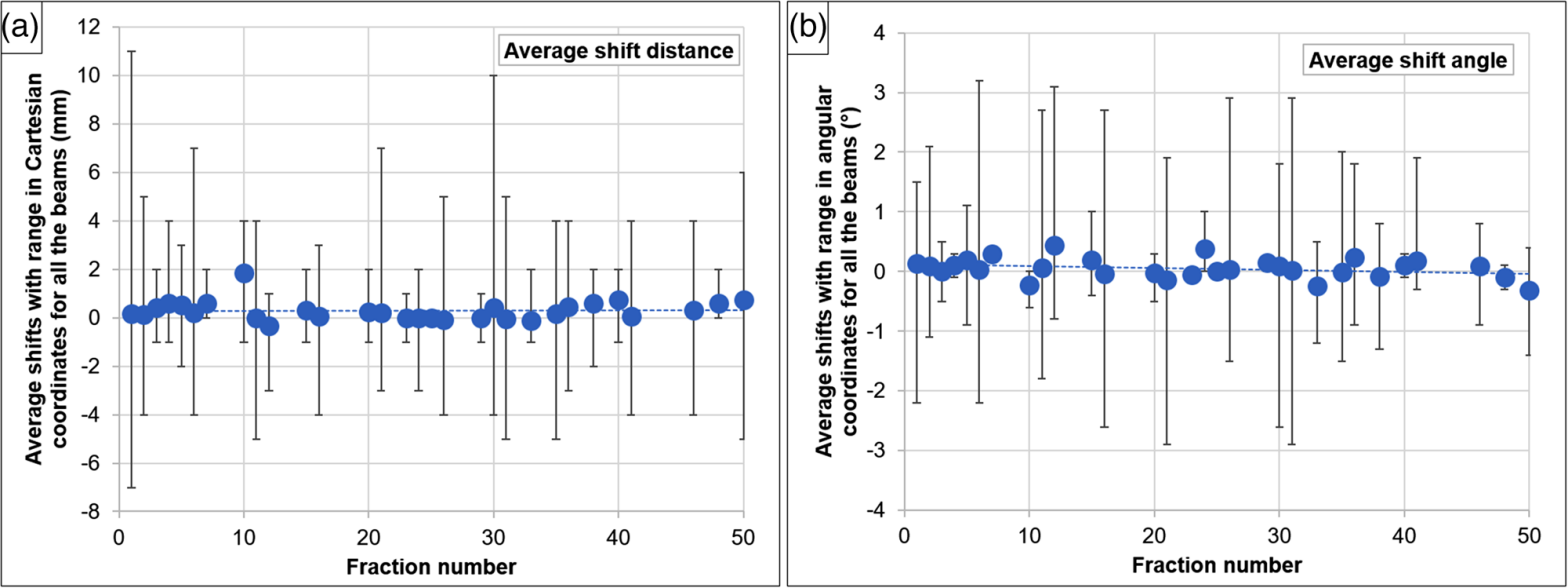
Average couch shifts (blue circles) and minimum–maximum range (vertical bars) for all beams and over all 28 patients at the respective fraction number: (a) Cartesian coordinates in mm, where blue dots represent the average of longitudinal, vertical, and lateral shifts; and (b) angular coordinates in degrees, where blue dots represent the average of pitch and roll shifts

Figure 3 shows the averaged couch shifts (Δ*r* and Δ*θ*) and their ranges over all beams for the 28 patients at the respective fractions. There was a shift increase toward the end of treatment courses for 17 patients. These 17 patients were treated with 30–35 fractions, once a day. This increase may be due to changes in patient weight and ill‐fitting masks that might have deteriorated during treatment.

**FIGURE 4:**
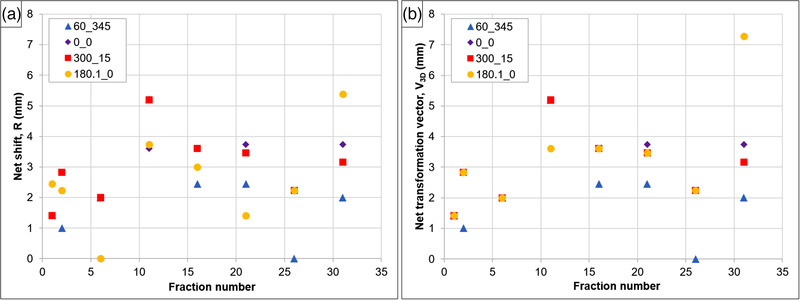
Couch shifts in a representative patient over the course of a 33‐fraction treatment: (a) Cartesian net shift; and (b) 3D transformation vectors *V_3D_* for each beam. Each beam is represented by the gantry (first number) and couch angles (second number) in degrees. The sequence of beams was delivered in an anticlockwise direction.

The net shift for a representative patient and associated net transformation vector are presented in Figure 4. The patient was a 69‐year‐old man diagnosed with a malignant neoplasm at the base of the tongue. The patient's body mass index (BMI) was 33.4 kg/m^2^ at treatment planning, and his weight decreased from 120.0 to 112.4 kg over the course of treatment. A dose of 70 Gy was delivered in 33 fractions, using 4 pencil‐beam scanning proton beams (2 coplanar and 2 noncoplanar beams). Couch shifts were recorded for first, second, and every fifth fraction thereafter. Out of 96 Cartesian coordinates, 42 were zero, and the remaining 54 were nonzero. Similarly, out of 64 angular coordinates, 22 were zero, and the remaining 42 were nonzero. For this patient, both the net couch shift and net transformation vector increased toward the end of the course of treatment, and both the net couch shifts and net transformation vectors were of similar values. For 17 of 28 patients, couch shifts were greater toward the end of the course of treatment.

**FIGURE 5:**
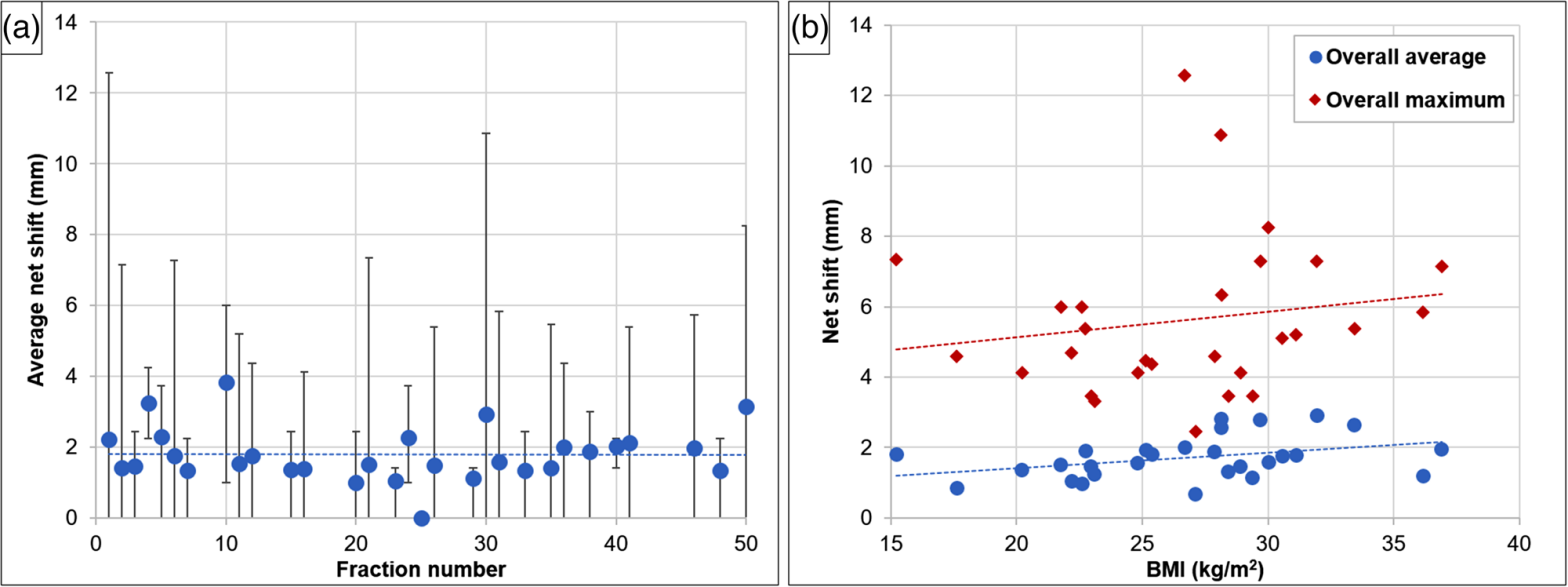
Net shift analysis for all 28 patients: (a) average net shifts (blue circles) and minimum–maximum range (vertical bars) for all beams and over all 28 patients at the respective fraction numbers; (b) overall averages (blue dots) and maximum (red squares) net shifts versus patient body mass index (BMI). Squared‐dotted lines represent data trend lines.

Out of all the couch data collected, a total of 1073 net shifts were noted for all 28 patients. As shown in Figure 5a, the net shifts varied between 0 and 12.6 mm. We averaged the net couch shifts over all the beams and for all the fractions (overall average) for each patient and plotted these against their BMIs. Figure 5b shows a trend toward increasing net couch shifts with increasing BMI. With BMI < 27 kg/m^2^, overall averages were <2 mm, and overall maximum net shifts were <6 mm, with a single exception of a 67‐year‐old man with BMI of 15.2 kg/m^2^ (lowest BMI of the 28 patients). In our study, both weight change and BMI contributed to the net shift of the couch. Weight change caused the mask to be ill‐fitting, and higher BMI caused more couch sag, with each contributing to greater couch shift.

## 4 DISCUSSION

Intrafraction setup uncertainty comes from both the 6‐DOF robotic couch and from the patient's status/well‐being on the day of treatment. The couch shifts shown in Figures 2, 3, 4, 5 are statistically significant and depend on several factors: the couch position tolerance from the machine (1 mm in Cartesian coordinates and 1° in angular coordinates from the daily and monthly QA), the patient's overall health status on the day of treatment (e.g., limited mobility), weight loss over the course of the treatment, BMI, and machine performance. Thus, any shift >1 mm was derived from the patient positioning uncertainty. When combining Cartesian shifts <2 mm, the net shift could be >2 mm. Therefore, these shifts should always be evaluated in a 3D manner to determine the dosimetric significance of the shifts. Both the net couch shift and net transformation vector showed similar values and trends along the course of treatment. Hence, the shifts observed at the couch system coordinates are true shifts and the same as those observed at beam's‐eye view. We do not consider the position‐dependent accuracy of our robotic couch, because we require IGRT for each beam unless the couch is not moved between the beams. Our results partially reflect position‐dependent variations. For the same reason, our QA does not check the accuracy at different positions.

Intrafractional changes could impact the accurate delivery of IMPT plans more severely than that of the IMRT plans. Muller et al.^12^ studied the impact of intrafractional changes in nonrobustly optimized H&N plans on the delivered dose in IMPT and IMRT. They found that tumor coverage was less sensitive for the photon plans and that the proton plan dose distributions indicated a high risk of undercoverage to the CTV. Muller et al. also reported an overdosage of OARs in IMRT plans due to intrafractional geometric changes. In our study, one patient's plan was recalculated by shifting the isocenter of the beams (2 mm in each direction) as found from the kV images during setup. From the calculated dose, it was found that the dose to 99% of the CTV was 3.4% lower than that in the original plan, and the dose outside the CTV could be as much as 13% higher than the actual plan. Therefore, at our center, it is essential to carefully evaluate the beams with imaging before delivering proton beam therapy (PBT) to bilateral H&N patients.

Because the couch shifts were random, we could not build up a prediction model to identify when couch shifts are required for setup. Thus, we propose that kV imaging should be performed for each field, upon couch rotation and before treatment delivery. The exception for this recommendation is for patients with BMI < 27 kg/m^2^ and when there is no weight loss over the course of the treatment; in such cases, one may skip the kV imaging followed by a couch rotation.

## 5 CONCLUSION

This retrospective study confirms the need at our center for orthogonal kV imaging for most patients upon couch rotation before delivering each field of bilateral H&N PBT. About 64.3% of the Cartesian shifts were zero and 35.7% non‐zero, ranging from −7 to +11 mm. For angular shifts, 48.2% were zero and 51.8% non‐zero, ranging from −3.0° to +3.2°. However, the overall Cartesian and angular shifts remained consistent across the treatment course. We also found a correlation between couch shift and BMI, with the net shift increasing with BMI. With BMI < 27 kg/m^2^, overall averages were <2 mm, and overall maximum net shifts were <6 mm. Further couch shift analyses are warranted to assess the correlation of couch shifts in different patient populations.

## AUTHOR CONTRIBUTION

Each author in this manuscript has contributed in one or more assignments; for example, idea development, data collection, data analysis, result discussions, manuscript writing, and editing.

## CONFLICT OF INTEREST

The authors have no conflicts of interest to disclose.

## Data Availability

The data that support the findings of this study are available from the corresponding author upon reasonable request.

## References

[1] Lin A , Swisher‐McClure S , Millar LB , et al. Proton therapy for head and neck cancer: current applications and future directions. Transl Cancer Res. 2012;1(4):255‐263.

[2] Hu M , Jiang L , Cui X , Zhang J , Yu J . Proton beam therapy for cancer in the era of precision medicine. J Hematol Oncol. 2018;11(1):136. 30541578 10.1186/s13045-018-0683-4 PMC6290507

[3] Frank SJ , Cox JD , Gillin M , et al. Multifield optimization intensity modulated proton therapy for head and neck tumors: a translation to practice. Int J Radiat Oncol Biol Phys. 2014;89(4):846‐853. 24867532 10.1016/j.ijrobp.2014.04.019 PMC4171724

[4] Kim JK , Leeman JE , Riaz N , McBride S , Tsai CJ , Lee NY . Proton therapy for head and neck cancer. Curr Treat Options Oncol. 2018;19(6):28. 29744681 10.1007/s11864-018-0546-9

[5] Iwata H , Toshito T , Hayashi K , et al. Proton therapy for non‐squamous cell carcinoma of the head and neck: planning comparison and toxicity. J Radiat Res. 2019;60(5):612‐621. 31147697 10.1093/jrr/rrz036 PMC6805978

[6] Beddok A , Vela A , Calugaru V , et al. Proton therapy for head and neck squamous cell carcinomas: a review of the physical and clinical challenges. Radiother Oncol. 2020;147:30‐39. 32224315 10.1016/j.radonc.2020.03.006

[7] Kraan AC , van de Water S , Teguh DN , et al. Dose uncertainties in IMPT for oropharyngeal cancer in the presence of anatomical, range, and setup errors. Int J Radiat Oncol Biol Phys. 2013;87(5):888‐896. 24351409 10.1016/j.ijrobp.2013.09.014

[8] Nath SK , Simpson DR , Rose BS , Sandhu AP . Recent advances in image‐guided radiotherapy for head and neck carcinoma. J Oncol. 2009;2009:752135. 19644564 10.1155/2009/752135 PMC2717698

[9] Mendenhall NP , Malyapa RS , Su Z , Yeung D , Mendenhall WM , Li Z . Proton therapy for head and neck cancer: rationale, potential indications, practical considerations, and current clinical evidence. Acta Oncol. 2011;50(6):763‐771. 21767172 10.3109/0284186X.2011.590147

[10] Stoiber EM , Bougatf N , Teske H , et al. Analyzing human decisions in IGRT of head‐and‐neck cancer patients to teach image registration algorithms what experts know. Radiat Oncol. 2017;12(1):104. 28637483 10.1186/s13014-017-0842-8 PMC5480194

[11] Ford EC , Evans SB . Incident learning in radiation oncology: a review. Med Phys. 2018;45(5):e100‐e119. 29419944 10.1002/mp.12800

[12] Muller BS , Duma MN , Kampfer S , Nill S , Oelfke U , Geinitz H , Wilkens JJ . Impact of interfractional changes in head and neck cancer patients on the delivered dose in intensity modulated radiotherapy with protons and photons. Phys Med. 2015;31:266‐272. 25724350 10.1016/j.ejmp.2015.02.007

[13] Ye JC , Qureshi MM , Clancy P , Dise LN , Willins J , Hirsch AE . Daily patient setup error in prostate image guided radiation therapy with fiducial‐based kilovoltage onboard imaging and cone‐beam computed tomography. Quant Imaging Med Surg. 2015;5(5):665‐672. 26682136 10.3978/j.issn.2223-4292.2015.10.01 PMC4671976

[14] Gu W , Neph R , Ruan D , Zou W , Dong L , Sheng K . Robust beam orientation optimization for intensity‐modulated proton therapy. Med Phys. 2019:46(8):3356‐3370. 31169917 10.1002/mp.13641 PMC6692214

[15] Tanaka Y , Oita M , Inomata S , Fuse T , Akino Y , Shimomura K . Impact of patient positioning uncertainty in noncoplanar intracranial stereotactic radiotherapy. J Appl Clin Med Phys. 2020;21(2):89‐97. 10.1002/acm2.12820 PMC7020988 31957975

[16] Sarkar B , Ray J , Ganesh T , et al. Methodology to reduce 6D patient positional shifts into a 3D linear shift and its verification in frameless stereotactic radiotherapy. Phys Med Biol. 2018;63(7):075004. 29480166 10.1088/1361-6560/aab231

[17] Huang Y , Zhao B , Kim J , Wen N , Chetty IJ , Siddiqui S . Targeting accuracy at couch kick for a frameless image guided radiosurgery system. J Radiosurg SBRT. 2018:5:123‐129. 29657893 PMC5893453

